# Next Generation Sequencing Technologies: The Doorway to the Unexplored Genomics of Non-Model Plants

**DOI:** 10.3389/fpls.2015.01074

**Published:** 2015-12-16

**Authors:** Chibuikem I. N. Unamba, Akshay Nag, Ram K. Sharma

**Affiliations:** ^1^Biotechnology Division, CSIR-Institute of Himalayan Bioresource TechnologyPalampur, India; ^2^Department of Plant Science and Biotechnology, Imo State UniversityOwerri, Nigeria

**Keywords:** non-model, genomics, next generation sequencing, whole genome, transcriptome

## Abstract

Non-model plants i.e., the species which have one or all of the characters such as long life cycle, difficulty to grow in the laboratory or poor fecundity, have been schemed out of sequencing projects earlier, due to high running cost of Sanger sequencing. Consequently, the information about their genomics and key biological processes are inadequate. However, the advent of fast and cost effective next generation sequencing (NGS) platforms in the recent past has enabled the unearthing of certain characteristic gene structures unique to these species. It has also aided in gaining insight about mechanisms underlying processes of gene expression and secondary metabolism as well as facilitated development of genomic resources for diversity characterization, evolutionary analysis and marker assisted breeding even without prior availability of genomic sequence information. In this review we explore how different Next Gen Sequencing platforms, as well as recent advances in NGS based high throughput genotyping technologies are rewarding efforts on *de-novo* whole genome/transcriptome sequencing, development of genome wide sequence based markers resources for improvement of non-model crops that are less costly than phenotyping.

## Genomics in the viewpoint of non-model plant systems

Plant genomics, which entails the application of recombinant DNA technologies, sequencing methods, and Bioinformatics tools for assembling and assigning the function and structure of plant genomes, is a key to understanding their genome via determining the order of DNA sequences which sequentially enable exploring the evolution of plant genome structure and inferring molecular phylogeny. It also helps in fathoming the interaction of genes in controlling the organism growth, development and adaptation to their environment. Most of the information we have about mechanisms underlying plant biological processes come from investigations on “model plants,” commonly referred to as “plants” extensively studied at the whole genome level to elucidate various complex biological phenomena. High-throughput sequencing technologies are, however changing the approaches toward projects geared at genome sequencing, giving us a deeper understanding of plant biology by the generation of biologically important data sets from different plant species other than the model plants.

Prior to the development of next generation sequencing (NGS) in 2005 (Morozova and Marra, 2008; Schuster, 2008), nucleic acid sequencing for genomic studies was based on the Sanger method. This technique was successfully used to complete the human genome and the first sequenced plant genome, *Arabidopsis thaliana (The Arabidopsis Genome Initiative)* published in 2000. The authors, after outlining its many advantages for genome analysis, which include small size, homozygous nature, large number of offspring due to short gestation period and relatively small nuclear genomes, reported the plant as an important model system for identifying pathways genes and determining their functions. Some other plants sequenced using this first generation method and reported by Schatz et al. (2012) as models include *Oryza sativa* (rice) in 2002, *Carica papaya* (papaya) in 2008 and *Zea mays* (maize) in 2009. Further genome sequencing of other plants was therefore based on the idea that a single species among related plant species that share some similarities is chosen as a model, studied as a representative and information gathered can be applied to related organisms as required but Tagu et al. (2014) noted that model organisms are often not archetypal and do not replicate the biology of their close relatives or even the wide diversity of living mechanisms. Hirsch and Buell (2013) stated that the characteristics of the ideal plant genome are hinged on technological limitations of genome-sequencing and assembly methods, computation, and the desire for a whole genome sequence for downstream biological interpretations. Despite the successful use of Sanger technology in sequencing the model crops, its throughput and high cost posed some constraints to sequencing millions of plant species, especially those with large and complex genomes and this prompted high demand for new and improved sequencing technologies. In addition, several non-model plants are indispensable assets for food, feed, or energy resource with certain characteristics unique to them and thus intricate to study them by the use of a model plant (Carpentier et al., 2008); hence, genomics in these species was not known and posed some challenges until the recent progress made by the emergence of alternative sequencing platforms with increased throughput and lower sequencing cost collectively termed as NGS technologies.

This article is therefore an appraisal of the impact made by NGS technologies on the “genomics of non-model plants.” It also tried to make out the future prospect of using these technologies in this group of plants.

## Glimpse of next generation sequencing technologies (NGS)

NGS incorporates technologies which at low cost and in short time produces millions of short DNA sequence read mostly in the range of 25 and 700 bp in length. According to Metzker (2010), they include a number of methods grouped broadly as template preparation, sequencing and imaging, and data analysis in which protocol distinguishes one technology from another and by the amount of the data produced from each platform. NGS has turned out to be a realistic method for maximizing sequencing in a large number of non-model plants while reducing time and cost when compared to the traditional Sanger method. The Sanger method makes use of the 2′,3′-dideoxy and arabinonucleoside analogs of the normal deoxynucleoside triphosphates, which act as specific chain-terminating inhibitors of DNA polymerase (Sanger et al., 1977) while in the NGS techniques, the DNA sequencing libraries are first clonally amplified *in vitro*, circumventing the time consuming and laborious cloning of the DNA library into bacteria unlike the Sanger method (Anderson and Schrijver, 2010). In addition, DNA templates are randomly read along the entire genome in a massively parallel sequencing by splitting the entire genome into small pieces followed by adapter ligation to the fragmented DNA (Zhang et al., 2011). Different technologies comprise NGS and while some of these technologies seem to have slight common features, they share key characteristics (Supplementary Table 1). The most commonly used platforms for high-throughput, useful genomic research, especially in non-model plant species include, Illumina/Solexa, 454/Roche, ABI/SOLiD, and Helicos. Results obtained from such research point to the fact that NGS techniques should not be restricted to the genomes of model organisms only as non-model plants have provided useful resources for genomic studies. Though they have their shortcomings, they are better off than the traditional Sanger method as shown in Supplementary Table 1.

## NGS enabled genomic research in non-model plants

### Whole genome sequencing

High coverage and quality reference genome sequences which give insight into the relatively complete information of genes, the regulatory elements that control their function, genome composition and an outline for understanding genomic variations (Feuillet et al., 2011) are the basics of “omics” investigations in a targeted species (Wei et al., 2013). The low cost of NGS is making it achievable for non-model plants, but as highlighted by Hirsch and Buell (2013), four major factors hinder the obtaining of quality genome assembly from non-model species: the extent of genome duplication (segmental, tandem, and whole-genome); the heterozygosity; the ploidy level; and repetitive sequence composition which have until now thwarted full genome sequencing and assembly of these plants. However, different methods are being applied to obtain a good quality sequence data as most sequencing projects of non-model plants are *de novo*, therefore, sequencing and assembly require high coverage and quality sequence data.

Various strategies are being employed to overcome the high level of heterozygosity and repetitive sequences that hinder the sequencing and assembly of plants using NGS technologies. Sequencing several independent libraries with different insertion sizes in different platforms and combining their data for assembly (Peng et al., 2014) wherein all data put together achieved high coverage of the genome and consequently enhanced the quality of the *de novo* assembly. Combined sequence data from paired end and mate pair libraries also produce assemblies with longer contigs and fewer, larger scaffolds for maximizing coverage across the genome, thus many biological questions in these non-model plants can be answered. The large genome size of these plants is contributed by highly repetitive sequences that are similar or identical to sequences in the genome, are so abundant in occurrence such that even sequencing to higher depths by short-read technologies does not guarantee assembly quality. According to Hirsch and Buell (2013), their overrepresentation in the read pool of short-read sequences when joined with the inherent error rate in current NGS technologies confounds genome assembly. However, a hybrid approach that combines WGS sequencing data from different short reads platforms with high-density genetic and physical maps was utilized by Kane et al. (2011); Yang et al. (2013); Chen et al. (2013) wherein the maps can serve as scaffolds for the linear assembly of WGS sequences. Heterozygosity hampers contig assembly when a whole-genome shotgun strategy is used for sequencing. The negative effect of ploidy level and heterozygosity to the assembly of short-read sequence can be cushioned using homozygous genotypes derived from successive generations of self-fertilization (Shulaev et al., 2011; Wang et al., 2012a; Polashock et al., 2014). Wu et al. (2013) employed a novel combination of BAC-by-BAC (bacterial artificial chromosome) libraries with Illumina sequencing technology and Liu et al. (2014) used BAC libraries successfully, to overcome the major issues of high heterozygosity and high repeat content. This showed that a complex plant genome sequence can be assembled and characterized using NGS without a physical reference.

Genome duplication is thought to be a factor in the evolution and diversification of plants. Whole genome duplication (WGD) creates gene duplicates in plants, some which might not be essential to cell functioning while some may evolve novel genes via non-functionalization, neofunctionalization, or subfunctionalization. WGD thus contributes to evolution by enabling the evolution of new gene functions, advancing genome rearrangement and perhaps driving speciation. Whole genome sequencing (WGS) and analysis methods by comparing the sequences of individual members of a family is helping to map out the individual gene duplications involved in the evolution of a family from a single progenitor gene that existed in an ancestral genome as seen in Albert et al. (2013) where genomic changes that accompanied the origin of angiosperms was identified. They showed an ancient genome duplication that predated angiosperm diversification indicating that the ancestral angiosperm was a polyploid with a large assemblage of both novel and ancient genes that survived to play key roles in angiosperm biology.

The complete genome sequence of a species nevertheless does not imply that all accessions of the species has the same nucleotide sequence but rather contains almost same set of genes with changes in their nucleotide sequence arising maybe from substitutions, insertions, deletions, and structural variations. The low cost of NGS has made sequencing of related genomes to estimate the genetic diversity within and between germplasm pools possible, and identification and tracking of genetic variation are now so efficient and precise that thousands of variants can be tracked within large populations (Varshney et al., 2009). In sequencing the genomic DNA and RNA of *Cannabis sativa* (Purple Kush) using hybrid approaches of Illumina and 454 pyrosequencing, Van Bakel et al. (2011) reported a draft haploid genome sequence of the cultivar which, when compared with the genome of another cultivar *C. sativa* (Finola), showed more expression of cannabinoid pathway genes and the exclusive presence of the functional THCA synthase (THCAS) in the genome and transcriptome of Purple Kush. Deciphering domestication of plants requires identification of the important traits that have been altered during domestication. NGS have made the discovery of the genes that have been selected during domestication feasible. Investigation of the primary gene pool and of more distantly related wild relatives has potential to identify genes and alleles that can be used to improve the performance of major crop species (Tang et al., 2010). Mace et al. (2013) used WGS to give an account of a strong racial structure and complex domestication events in 44 accessions of Sorghum and showed that the modern cultivated sorghum is derived from a limited sample of racial variation, with the result pointing to the positive utilization of NGS in the understanding of genetic diversity at the genomic sequence level.

To date, a number of non-model crops have been successfully sequenced using the NGS technology (Table 1) charting a new course for future genomic and genetic research and crop improvement in these plants, and even turning some of the so called non-model plants into genetic models for studying certain biological processes.

**Table 1:** **Non-model plants sequenced using next generation sequencing technology**.

**S/N**	**PL**	**CN**	**EGS**	**SP**	**SC**	**AGS**	**AGC (%)**	**SN50**	**CN50**	**NG**	**GSO**	**REFERENCES**
1	*Solanum commersonii*	Commerson's nightshade	840 Mbp	I	105x	838 Mb	98	44.29 Kb	6.5 Kb	37,662	The draft genome sequence of *S. commersonii* substantially increases our understanding of the domesticated germplasm, facilitating translation of acquired knowledge into advances in crop stability in light of global climate and environmental changes.	Aversano et al., 2015
2	*Gossypium hirsutum* TM-1	Cultivated cotton	2.25–2.43 Gb	I	181x	2.1 Gb	96.70	107 kb	20 kb	76,943	The complex genome of *Gossypium hirsutum* has been elucidated in this study, which was proven difficult to sequence owing to its complex allotetraploid (AtDt) genome.	Li et al., 2014
3	*Conyza canadensis*	Horseweed	335 Mb	R, I and PB	~350×		92.30	33.5 Kbp	20.8 kbp	44,592	Reportedly the first published draft genome of an agricultural weed which is a useful genomic resource for understanding weediness and the evolution of herbicide resistance as well as development of control strategies.	Peng et al., 2014
4	*Vaccinium macrocarpon*	American cranberry	470 Mbp	I	20x	420 Mbp	93	4.2 kbp		36,364	The study demonstrated the use of an inbred genotype derived from five generations of selfing to reduce heterozygosity and identified candidate genes which will be useful for further studies on biochemical pathways and cellular processes as well as development of molecular markers for breeding.	Polashock et al., 2014
5	*Ziziphus jujuba*	Jujube	444 Mb	I	429.25x	437.65 Mb	98.60	301.04 kbp	33.95 kbp	32,808	The study provides insights into jujube-specific biology and valuable genomic resources for the improvement of Rhamnaceae plants and other fruit trees.	Liu et al., 2014
6	*Solanum melongena*	Eggplant	1.1 Gb	I and R		833 Mb	74	64 kbp		85,446	The study gave an insight into the eggplant genome structure and will be a milestone for understanding unexplored species of the Solanaceae.	Hirakawa et al., 2014
7	*Humulus lupulus*	Hops	2.57 Gb	I	164x	2.05 Gb	80	37 kbp		41,228	The study which utilized two cultivars suggested the significance of historical human selection process for enhancing aroma and bitterness biosyntheses in hop cultivars, and as well serve as crucial information for breeding varieties with high quality and yield.	Natsume et al., 2014
8	*Camelina sativa*	False flax	785	I and R	123x	641.45 Mb	82	30.09 Mb	33.41 Kb	89,418	The study provides first chromosome-scale high-quality reference genome sequence for *C. sativa*, representing a whole-genome triplication event relative to the crucifer model *Arabidopsis thaliana*.	Kagale et al., 2014
9	*Oryza glaberrima*	African Rice		R and S		316 Mb		217 kb		33,164	This study provides evolutionary history of domestication and selection in African rice and supports the hypotheses that, it was domesticated in a single region, as opposed to domestication events across Africa.	Wang et al., 2014a
10	*Oryza sativa AA genom*	Wild rice		I							The WGS among the closely related wild rice species in different continents gave insight into plant gene and genome evolution. The study identified genomic variations, including segmental duplication and diversifying natural selection. It also indicated specific genes responsible for the adaptations.	Zhang et al., 2014
	*-Oryza nivara*		395 Mb		~73x	375 Mb	94.90	511.54 kbp	19.02 bp	41,490	
	*-Oryza glaberrima*		370 Mb		~56x	344 Mb	93.20	722.13 kbp	25.248 bp	41,476	
	*-Oryza barthii*		376 Mb		~51x	335 Mb	89.10	237.57 kbp	16.126 bp	41,605	
	*-Oryza glumaepatula*		366 Mb		~86x	344 Mb	91.40	129.69 kbp	17.474 bp	39,106	
	*-Oryza meridionalis*		388 Mb		~60x	340 Mb	87.8	117.67 kbp	14.633 bp	42,283	
11	*Pinus taeda*	Loblolly pine	20.15 Gb	I	98%			66.9 kbp		50,172	In this study, the large genome of the Loblolly pine (≈ 20–40 Gb, 2*n* = 24) has been annotated for the first time by whole-genome shotgun assembly which comprises 20.1 Gb of sequence.	Wegrzyn et al., 2014
12	*Spirodela polyrhiza*	Greater duckweed	158 Mbp	R and S	5x			3.76 Mb		19,623	In this study, it has been observed that, *Spirodela* has a genome with no signs of recent retrotranspositions but signatures of two ancient whole-genome duplications, possibly 95 million years ago (mya), older than those in Arabidopsis and rice. Its genome has only 19,623 predicted protein-coding genes, which is 28% less than the dicotyledonous *Arabidopsis thaliana* and 50% less than monocotyledonous rice.	Wang et al., 2014b
13	*Capsicum annuum*	Pepper	3.48 Gb	I and S	186.6x	3.06 Gb	90				WGS of *Capsicum annuum* integrated with data from resequencing of two cultivated peppers and *de novo* sequencing of a wild species afforded an evolutionary view into the genome expansion, origin of pungency, distinct ripening process and disease resistance of *Capsicum annuum* and gave an insight to the capsaicinoid pathway.	Qin et al., 2014; Kim et al., 2014
	cv. *CM334*							2.47 Mb	30.0 kb	34,903		
	cv. *Zunla-1*							1.23 Mb	55.4 kb	35,336		
14	*Amborella trichopoda*	Amborella	870 Mb	R and I	~30x	706 Mb	81	4.9 Mbp		26,846	Study showed an ancient genome duplication preceding angiosperm diversification providing basis for understanding major genomic events in angiosperm evolution including polyploid origin of angiosperms and hexaploidization event in eudicots.	Albert et al., 2013
15	*Lupinus angustifolius*	Lupin	1.153 Gb	I	26.9x	598 Mbp	51.90	12.5 kbp	5.8 kbp	57,807	The study demonstrated the cost effectiveness of NGS in generating genomic resources for genomic and genetic studies in lupin and other non-model plants by combination of medium-depth genome sequencing and a high-density genetic linkage map.	Yang et al., 2013
16	*Oryza brachyantha*	Wild rice	~297 Mb	I	~104x	262 Mb	96	1.0 Mbp	20.4 kbp	32,038	The high-quality reference genome sequence of *Oryza brachyantha* provides an important resource for functional and evolutionary studies in the genus *Oryza*.	Chen et al., 2013
17	*Pyrus bretschneideri*	Pear	527 Mb	I	194x	512 Mb	97.10	540.8 kbp	35.7 kbp	42,812	The WGS of the plant in addition to providing an invaluable new resource for biological research of *Pyrus* gave insights into mechanisms underlying important biological processes, including stone cell formation, sugar accumulation, and aroma formation and release.	Wu et al., 2013
18	*Nelumbo nucifera*	Sacred lotus	929 Mb	I/R	101/5.1x	804 Mb	86.50	3.4 Mbp	38.8 kbp	26,685	WGS reported a lineage-specific duplication in *Nelumbo nucifera* and lack of triplication event, characteristic of other eudicots thus making it a model for reconstructing the pan-eudicot genome and comparing eudicots and monocots.	Ming et al., 2013
19	*Genlisea aurea*	Corkscrew plant	63.6 Mb	I		43.4 Mb	68		5.78 kbp	17,755	Identified low number of genes despite being a carnivorous plant but introns and intergenic regions are unusually short and observed that reduction of genome size in the *G. aurea* lineage was due to both gene loss and non-coding sequences shrinking, but not to intron loss.	Leushkin et al., 2013
20	*Betula nana*	Dwarf birch	448 Mb	I	66x			18.6 kbp	5 kbp		The work presented a preliminary study of allele sharing among species, demonstrating the utility of the data for introgression studies and for the identification of species-specific alleles.	Wang et al., 2013
21	*Actinidia chinensis*	kiwifruit	758 Mb	I	140x	616.1 Mb	81.30	646.8 kbp	58.8 kbp	39,040	The study revealed WGD events undergone by the plant, detected heterozygous sites revealing high level of heterozygosity of the plant while providing a valuable resource for biological discovery, crop improvement and comparative genomic analysis.	Huang et al., 2013
22	*Hevea brasiliensis*	Rubber	~2.15 Gb	I, R and S	~43x	~1.1 Gb		3 kbp		68,955	The WGS in addition to key genes associated with rubber biosynthesis, rubberwood formation, disease resistance, and allergenicity identified a higher percentage of repetitive sequences which posed a challenge to the whole genome assembling.	Rahman et al., 2013
23	*Citrullus lanatus*	Water melon	~425 Mb	I	108.6x	353.5 Mb	83.20	2.38 Mb	26.38 kb	23,440	The WGS identified genomic regions that were preferentially selected as well as many disease-resistance genes lost during domestication in addition to providing insights into aspects of phloem-based vascular signaling in common between watermelon and cucumber and identification of genes crucial to valuable fruit-quality traits, including sugar accumulation and citrulline metabolism.	Guo et al., 2013
24	*Triticum urartu*	Einkorn wheat	4.94 Gb	I	~91x	4.66 Gb	94	63.69 kbp	3.42 kbp	34,879	The genome assembly provides a diploid reference for analysis of polyploid wheat genomes and is a valuable resource for the genetic improvement of wheat.	Ling et al., 2013
25	*Beta vulgaris*	Sugar beet	714–758 Mbp	I, R, and S	93x	569.0 Mb		2.01 Mb	1.7 Mb	27,421	Phylogenetic analyses in this study provided evidence for the separation of Caryophyllales before the split of asterids and rosids, and revealed lineage-specific gene family expansions and losses.	Dohm et al., 2014
26	*Gossypium raimondii*	Cotton		I	103.6x	775.2 Mb	88.10	2284 kbp	44.9 kb	40,976	The study observed evidence of the hexaploidization event shared by the eudicots as well as of a cotton-specific whole-genome duplication ~13–20 MYA.	Wang et al., 2012a
27	*Linum usitatissimum*	Flax	373 Mb	I	94x	318 Mb	85	694 kbp	20.1 kbp	43,384	The results from the demonstrated that *de novo* assembly of whole-genome shotgun short-sequence reads is an efficient means of obtaining nearly complete genome sequence information for some plant species.	Wang et al., 2012b
28	*Prunus mume*	Chinese Plum/Mei	280 Mb	I	101x	237 Mb	84.60	577.8 Kbp	31.8 Kbp	1154	The *P. mume* genome sequence contributes to the understanding of Rosaceae evolution and provided essential data for improvement of fruit trees.	Zhang et al., 2012
29	*Cyanophora paradoxa*	Glaucophyte	70 Mb	I, R, and S		70.2 Mbp			2.7 Kbp		In this study, analyses of the draft genome and transcriptome data from the basally diverging alga *Cyanophora* paradoxa was done and evidence for a single origin of the primary plastid in the eukaryote supergroup Plantae was established.	Price et al., 2012
30	*Bathycoccus prasinos* BBAN7	Green algae	15 Mb	S	22x	15.1 Mb				7847	The minimal genomes of the Mamiellophyceae provide a baseline for evolutionary and functional analyses of metabolic processes in green plants.	Moreau et al., 2012
31	*Chondrus crispus*	Red algae	105 Mb	S	14x	104.8		240 kb	64 kb	9606	Genome sequence of economically important red sea weed has been reported in this study.	Collén et al., 2013
32	*Cannabis sativa*	Cannabis	~820 Mb	I and R	110x	787 Mb	96	16.2 kbp		30,074	The study is an aid to the development of therapeutic marijuana strains with tailored cannabinoid profiles and provides a basis for the breeding of hemp with improved agronomic characteristics.	Van Bakel et al., 2011
33	*Fragaria vesca*	Woodland strawberry	~240 Mb	R, I, and S	39x	209.8 Mb	95%	1.3 Mb		34,809	WGS of fourth-generation inbred line of the *F. vesca* demonstrated that NGS can solely be used for assembling and characterization of a contiguous plant genome sequence while reporting the lack of large genome duplications seen in other rosids in the plant's sequence.	Shulaev et al., 2011
34	*Phoenix dactylifera*	Date palm	~658 Mb	I		381 Mb	~60	30.48 kbp		28,890	The study identified a region of the genome linked to gender and found evidence that date palm employs an XY system of gender inheritance.	Al-Dous et al., 2011

*PL, Plant Name; CN, Common Name; EGS, Estimated Genome size; SP, Sequencing Platform; SC, Sequencing Coverage; AGS, Assembled Genome Size; AGC, Assembly Genome Coverage; SN50, Scaffold N50; CN50, Contig N50; NG, Number of Genes; GSO, Genome Sequence Outcome; I, Illumina/Solexa; R, 454/Roche; S, SOLiD/ABI; H, Helicos; PB, Pac Bio System*.

## Gene identification and expression analysis

The field of molecular and evolutionary biology are being revolutionized by the accessibility to genome-scale information which has helped to answer biological questions like how the identical genetic makeup of cells can give rise to different cell types, with each playing a different role in the working of a multicellular organism that until recently were implausible. Earlier technique used for detection and quantification of specific RNA levels is the Northern blotting (Northern hybridization) developed by James Alwine and George Stark. In this technique, electrophoretically separated bands of RNA are transferred from an agarose gel to a paper strip. Specific RNA bands can be detected by hybridization with ^32^P-labeled DNA probes followed by autoradiography. This procedure allows the detection of specific RNA bands with high sensitivity and low background (Alwine et al., 1977). But as noted by Streit et al. (2008), northern blotting has some disadvantages among which are risk of mRNA degradation during electrophoresis, which compromises the quality and quantification of expression; health and environmental implication of high doses of radioactivity and formaldehyde; low sensitivity of northern blotting in comparison with that of RT-PCR; detection with multiple probes is difficult; use of ethidium bromide, DEPC and UV light needs special training and attention. The RNase protection assay, an alternative, is a highly sensitive technique developed to detect and measure the abundance of specific mRNAs in samples of total cellular RNA (Ma et al., 1996). Another method of gene expression analysis, hybridization of antisense RNA corresponding to a known complementary target sequence prevents target digestion by single strand–specific RNase activity. This process results in the degradation of all remaining single-stranded RNAs (i.e., those not hybridized to the probe sequence), enabling the accurate quantitation of specific target sequences (VanGuilder et al., 2008). However, the complex procedures as well as relatively large amounts of RNA involved pose some restrictions in the use of these methods. The development of real-time qPCR has increased the throughput of gene expression while reducing the required quantity of RNA. It has become a routine approach for measuring the expression of genes of interest, validating microarray experiments, and monitoring biomarkers (VanGuilder et al., 2008). Real-time PCR amplifies a specific target sequence in a sample, then monitors the amplification progress using fluorescent technology (Valasek and Repa, 2005). Despite the fact that real-time PCR technology is an invaluable tool for many scientists in gene expression analysis, its one major shortcoming is the prerequisite for prior sequence data of the specific target gene of interest, hence q-PCR can only be used for targeting of known genes.

The transcriptome is the set of all RNA molecules (mRNA, rRNA, tRNA, and other non-coding RNA) transcribed by an organism. Wang et al. (2009) had posited that the fundamental principle for interpreting the functional elements of the genome and revealing the molecular constituents of cells and tissues, and also for understanding development and disease is gaining insight into the transcriptome. Microarray is a technique widely employed for analyzing the transcriptome for patterns of gene expression. It has the ability to measure the expression levels of thousands of genes in a single experiment, but lacks the capacity to detect novel transcripts and sensitivity to expression levels of genes. NGS have rapidly advanced next-generation RNA sequencing (RNA-seq) for rapid generation of large expression datasets for gene discovery and expression analysis in non-model species (Marioni et al., 2008; Li et al., 2012). As stated by De Wit et al. (2012), RNA-seq focuses on sequencing only mRNA from the genes that are expressed in the tissue or transcriptome wherein a considerable proportion of adaptively interesting variations are located. It shows a record of how many mRNAs from a particular exon are in the sample and includes variations in the sequences that elucidate functional polymorphisms. Unlike the microarray techniques, RNA-seq can assemble reads *de novo* without mapping to reference genomic sequence, a feature that makes it an invaluable asset for identification of novel genes in non-model plants. Zhou et al. (2012) demonstrated the use of *de novo* assembly in *Ammopiptanthus*, a genus with evergreen broadleaf habit in the desert and arid regions of the Mid-Asia, playing a critical role in conserving the desert ecosystems, which is critical in controlling desertification. To understand the genetic mechanisms underlying deep, flourishing root system for water absorption to adapt these plants to harsh conditions, *de novo* transcriptome sequencing of *A. mongolicus* was carried out using 454 pyrosequencing to discover putative genes associated with drought tolerance. The potential drought stress related transcripts identified in the study provided a foundation for further investigation into the drought adaptation in *Ammopiptanthus*. Transcriptome sequencing has, however caused a significant upshot in the expressed sequence tags (ESTs) collections, including the non-model plant species (http://www.ncbi.nlm.nih.gov/dbEST/dbEST_summary.html).

MicroRNAs (miRNA; 21–24 nucleotide) are a class of non-coding endogenous small RNAs that are transcribed from a gene, but the transcript is never translated into a protein (Phelps-Durr, 2010) therefore are involved in regulating gene expression in different organisms including non-model plants. Since the discovery of the first miRNAs, *Lin-4*, Lee et al. (1993), there has been an increased interest in understanding post transcriptional gene expression regulation during development. According to Axtell and Bartel (2005), miRNAs affect the morphology of flowering plants by the post transcriptional regulation of genes involved in critical developmental events. They, however postulated that an understanding of the spatial and temporal dynamics of miRNA activity is fundamental to elucidate the functions of miRNAs. Achard et al. (2004) described the role of microRNA (miR159) in the regulation of short-day photoperiod flowering time and of anther development. Other plant developmental processes involving miRNAs include leaf morphogenesis and polarity (Floyd and Bowman, 2004), floral development and timing defects (Aukerman and Sakai, 2003) among others. Zhang et al. (2006) identified four existing approaches for identifying miRNAs which include genetic screening, direct cloning after isolation of small RNAs, computational strategy, and ESTs analysis but observed that these approaches have different advantages and shortcomings and postulated that combining these methods, more miRNAs will be quickly discovered. As reported by Lakhotia et al. (2014), a large number of miRNAs are evolutionary conserved among diverse species, while several miRNAs, that are considered to be recently evolved show species-specificity and often express at lower levels relative to conserved miRNAs and as a result of their low expression levels, most of the species-specific miRNAs remained unidentified in many plant species. With improved methods of NGS technologies in investigating the transcriptome, enormous progress, especially with regard to regulatory pathways have been made in identifying and understanding non-coding RNAs such as miRNAs. RNA sequencing using high-throughput NGS platforms has the advantage of high accuracy in distinguishing miRNAs that are very similar in sequence and can detect novel miRNAs. Gao et al. (2015) identified 50 novel miRNAs, representing 19 families from three sRNA libraries of tobacco in addition to 165 miRNAs representing 55 conserved families using Solexa sequencer. Similarly, using high-throughput sequencing of small RNAs and analysis of transcriptome data, Zhu et al. (2013), identified 132 putative conserved miRNAs belonging to 31 known miRNA families and 10 novel miRNAs in *Caragana intermedia*. They in addition, predicted 38 potential targets for the conserved and novel miRNAs and validated four of them by 5′ RACE. These including identifications of miRNA in various non-model crops, Lakhotia et al. (2014) show the value of high throughput sequencing approach to miRNA discovery, especially novel miRNAs in non-model crops without a reference genome.

## NGS in aid of molecular marker development and breeding

Molecular markers are identifiable DNA sequences, found at specific locations of the genome, and transmitted by the standard laws of inheritance from one generation to the next (Semagn et al., 2006). With the need to amplify the agricultural output to meet up with the challenge of producing enough food for the rising world population, advances in genomic technologies have provided new tools for discovering and tagging novel alleles and genes. These tools can enhance the efficiency of breeding programs through their use in marker-assisted selection (MAS), linkage mapping or quantitative trait locus (QTL) mapping, Phylogenetics, positional cloning, genetic diversity assessment, genotypic profiling etc. According to Kumpatla et al. (2012), the ability to deduce the underlying molecular mechanisms of a trait, understand the gene regulatory mechanisms, determine gene expression differences and variations in expressed gene sequences, and other structural variations such as copy number variations (CNV) and presence-absence variations (PAV) is to a large extent dependent on the availability of reference genome/transcriptome sequence.

Identification of polymorphic sequences, basic to a trait of interest enables the development of functional markers. The advent of NGS has enabled the exploration of thousands of markers across the entire genome using several approaches, enabling comprehensive genome-wide association studies, even in populations with little or any previous genetic information as in non-model plants (Sakiyama et al., 2014). SNP markers are the most abundant in a genome and appropriate for analysis on a wide range of genomic scales. SNPs are markers, which untangle polymorphism between individuals or populations due to change of a single nucleotide. Illumina transcriptome sequencing data was used to discover 2987 high-quality putative SNP in Turkish Olive Genotypes (Kaya et al., 2013). These were successfully used to access genetic diversity among 96 olive genotypes. A whole-genome resequencing of two cabbage inbred lines using Illumina (Lee et al., 2015) identified 674,521 SNPs. From these, 167 dCAPS markers were developed for genetic map construction which identified novel QTLs for black rot resistance. Similarly, a high-throughput and specific-locus amplified fragment sequencing (SLAF-seq) approach was also used by Wei et al. (2014a) to construct a high-density SNP map for cucumber. It contained 1800 high quality SNPs, spanning 890.79 cM with an average marker interval of 0.50 cM and further detected fruit-related QTLs. Also, genotyping-by-sequencing (GBS) approach via NGS identified 21,471 SNPs in oil palm (Pootakham et al., 2015). It enabled the construction of linkage map containing 1085 markers distributed over 17 linkage groups and identified quantitative trait loci (QTL) affecting trunk height and bunch weight.

Simple sequence repeat (SSR) markers which have the advantage of high abundance, random distribution within the genome, high polymorphism information content and co-dominant inheritance have been developed at large scale and lower costs via NGS. In *Myrica rubra* with an estimated genome size of 323 Mb, highly heterozygous but with little duplication, Jiao et al. (2012) identified 28,602 SSRs from a WGS sequencing using Illumina. Polymorphic markers among these also successfully transferred to other Myrica species. Likewise, in Sesame genome, 23,438 putative SSRs were identified by whole-genome *de novo* sequencing and successfully used to screen accession across 12 countries (Wei et al., 2014b). *De novo* genic SSRs have been developed at large scale and used in a number of non-model, including but not limited to *Caragana korshinskii* Kom (Long et al., 2015), *Hevea brasiliensis* (Salgado et al., 2014), *Prosopis alba* (Torales et al., 2013).

These developed markers are also used for association mapping studies in non-model plants. Association mapping (linkage disequilibrium mapping) identifies QTLs that accounts for phenotypic variation among individuals or species. It helps in the dissection of complex genetic traits and enhances crop breeding for traits as disease resistance, salinity and drought tolerance. In an association mapping analyses, accounting for population structure study by Gupta et al. (2014), eight out 50 SSR markers representing the nine chromosomes of foxtail millet used in testing population structure in 184 accessions were shown to have significant association with nine agronomic traits. Also, association analysis using 20 SSR markers to detect the marker loci linked to morphological traits and physiological traits in a wild *Populus simonii* population Wei et al. (2014c), revealed that three SSR markers were identified for seven traits, one was associated with five morphological traits while two of the markers were associated with one morphological trait and one physiological trait, respectively. These studies infer that the identified markers are suitable for MAS breeding, target gene detection or QTL.

Genome sequencing have aided in deciphering the influence of transposable elements in the function and evolution of genes and genomes. Most of these repetitive sequences are found in different regions across the genome and have been implicated in genome diversity and phenotypic variation. In view of these, molecular markers are being developed from these elements and used for diversity characterization and construction of genetic linkage maps. In foxtail millet, genome-wide analysis, Yadav et al. (2014) identified 30,706 TEs, which led to the development of 20,278 TE-based markers from namely Retrotransposon-Based Insertion Polymorphisms (4801), Inter-Retrotransposon Amplified Polymorphisms (3239), Repeat Junction Markers (4451), Repeat Junction-Junction Markers (329), Insertion-Site-Based Polymorphisms (7401) and Retrotransposon-Microsatellite Amplified Polymorphisms (57). Of these, 30 out of 134 Repeat Junction Markers screened in 96 accessions of *Setaria italica* and three wild *Setaria* accessions showed polymorphism. This demonstrates that transposable elements can serve as genomic resources for genotyping. Insertions and Deletions (Indels), are other genomic resources distributed across the genome that can also be used as molecular markers for Phylogenetics. 2687 InDel-based markers were developed from Illumina sequence data from three genotypes of *Phaseolus vulgaris* L (Moghaddam et al., 2014). These markers were successfully used to construct a phylogenetic tree and a genetic map, deducing that InDel markers are reliable, simple, and accurate. Introns are non-coding RNA transcripts that are spliced out before the translation of the RNA molecule into a protein. Markers developed from introns have high evolutionary rate, possibly because they are flanked by exons which consign conserved primers that may function across a wide range of species. Intron Length Polymorphic (ILP) markers are thus designed via exon-primed intron-crossing PCR (EPIC-PCR) by designing primers in exons flanking the target intron. NGS sequence data from a potato cultivar was used to design ILP markers (Ahmadvand et al., 2014). These markers were used to test diversity in other potato genotypes and cross transferability was investigated in other *Solanum* species. The results demonstrated ILPs as genomic resources in diverse molecular analyses, including cross-species studies. Similarly, Muthamilarasan et al. (2014) developed 5123 ILP markers, of which 4049 were physically mapped onto nine chromosomes of foxtail millet. They further showed the applicability of the markers in germplasm characterization, transferability, Phylogenetics and comparative mapping studies in millets and bioenergy grass species.

## Understanding biosynthetic pathways of specialized plant metabolites in non-model plants

Plants manufacture a huge and diverse group of organic compounds called secondary metabolites. These compounds appear to have no direct role in growth and other physiological processes in plants, but are implicated in their adaptation to their environment such as control of seed germination, symbiosis regulation, defense against herbivores and pathogens, and chemical inhibition of competing plant species. Contrasting the primary metabolites (sugars, amino acids, acyl lipids, and nucleotides) which are found in all plants, these secondary metabolites only pertain to a plant species or group of related plant species. They were initially thought to be waste products of metabolism until research showed that these secondary metabolites are useful in pharmaceuticals, flavors, industrial materials, and chemicals consequently increasing interest for their use. Most of these compounds occur in non-model plants for which genomic sequence information is not yet available (Xiao et al., 2013). The genus *Panax*, for instance, consists of at least nine species (Leung and Wong, 2010), most commonly referred to as ginsengs which are known from research to have anticancerous, antidiabetic, immunomodulatory, anti-inflammatory, and antiallergic, effects among other medicinal uses. The mode of action of ginseng was however not known until ginsenosides were isolated in 1963 (Shibata et al., 1963, 1965). Christensen (2008) reported that ginsenosides are found nearly exclusively in *Panax* species (ginseng) with more than 150 naturally occurring ginsenosides being isolated from the roots, leaves/stems, fruits, and/or flower heads of ginseng. Since then, research effort on evaluating the function and elucidating the molecular mechanism of each ginsenoside has been on the increase. Researchers have generated genomic information about ginsengs, identifying several candidate genes encoding enzymes responsible for the biosynthesis of the secondary metabolites ginsenoside using different NGS platforms (Sun et al., 2010; Luo et al., 2011; Li et al., 2013; Jayakodi et al., 2014).

Access to some of these secondary metabolic compounds was often poor because of a lack of understanding of how these metabolites are synthesized (Oksman-Caldentey and Inzé, 2004), partly owing to the fact that the enzymes and biochemical pathways in their synthesis were either unknown or having complexities that make identification of the enzymes that catalyze the numerous metabolic cycles difficult. In some of these plants, a number of regulatory enzymes are involved in the biosynthesis process. Many of the genes in plant genomes code enzymes for secondary metabolism and transcriptomics data mining however have proven to be an efficient way to discover genes or gene families encoding enzymes involved in various metabolic pathways (Xiao et al., 2013). *Podophyllum* species are sources of podophyllotoxin, an aryltetralin lignan used for semi-synthesis of various powerful and extensively employed cancer-treating drugs, its biosynthetic pathway, however, remains largely unknown. NGS/Bioinformatics and metabolomics analysis of *Podophyllum hexandrum* and *P. peltatum* plant tissues gave two putative genes in podophyllotoxin biosynthesis (Marques et al., 2013). Further studies using integrated omics technologies (including advanced mass spectrometry/metabolomics, transcriptome sequencing/gene assemblies, and Bioinformatics) in the two *Podophyllum* plants (Marques et al., 2014) enabled discovery of the aporphine alkaloid pathway in Podophyllum species, result which suggest evolutionary linkages between both lignan and alkaloid biosynthetic pathways. The authors reported that RNA-seq transcriptome sequencing and Bioinformatics guided gene assemblies/analyses *in silico*, specifically suggested presence of transcripts homologous to genes encoding all known steps in aporphine alkaloid biosynthesis. Miettinen et al. (2014) had stated that the biotechnological production progress of the monoterpenoid indole alkaloids (MIAs), produced by *Catharanthus roseus* in extremely low levels and used as anticancer drugs, from other sources is hampered by the lack of knowledge of the enzymes responsible for their biosynthesis. They nevertheless reported the characterization of the last missing steps of the *C. roseus* secoiridoid pathway using an integrated transcriptomics and proteomics approach for gene discovery, followed by biochemical characterization of the isolated candidates and further reported the reconstitution of the entire MIA pathway up to strictosidine in the plant host *Nicotiana benthamiana*, by heterologous expression of the newly identified genes in combination with the previously known biosynthesis genes. This new technology of NGS has helped in explicating the progression of events that lead to the production of these secondary compounds of interest in non-model plants, accelerating gene discovery for secondary metabolite pathways without preexisting sequence knowledge of the genes studied.

Many secondary metabolites have a complex and unique structure and their production is often enhanced by both biotic and abiotic stress conditions (Dixon, 2001). Ryan et al. (2002) provided valuable insights into the biochemical response of plants to UV stress, which results in the production of a more protective flavonoid profile. Rezaeieh et al. (2012) however noted that biotic and abiotic stresses exert an outstanding influence on the biosynthesis of several secondary metabolites in medicinal plants. Often, it is difficult to predict the complex signaling pathway that are activated or deactivated in response to different abiotic stresses but the complex molecular regulatory system involved in stress tolerance and adaptation in plants can be easily deciphered with the help of different omics study (Chawla et al., 2011). In response to various abiotic stresses, plant continuously needs to adjust their transcriptome profile (Gupta et al., 2013) thus NGS based transcriptome shotgun sequencing (RNA-seq), which targets the genes that are expressed in a tissue at a particular time is invaluable. A comprehensive transcriptome analysis of a salinity tolerant *Phaseolus vulgaris* L. variety by Illumina sequencing showed genes related to salt tolerance in plant (Hiz et al., 2014). This and other studies using transcriptomic approaches in non-model plants (Xu et al., 2013) for drought stress in *chrysanthemum* have continued to generate functional genomics resource, giving an unfathomable understanding of the molecular mechanisms underlying plant's responses to stress conditions.

## Conclusion and future prospects

**Figure 1:**
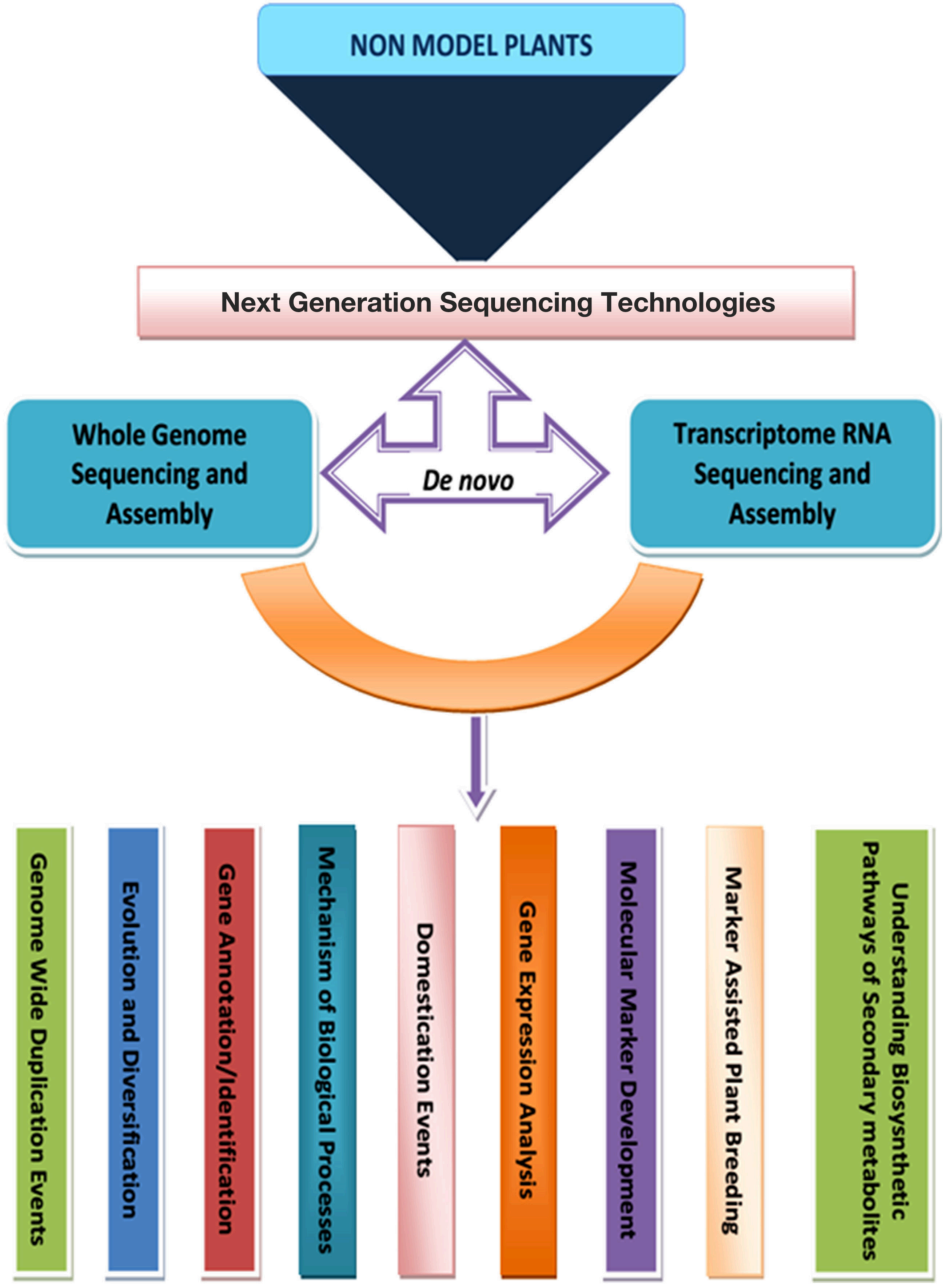
**Flow chart of NGS enabled genomic analysis in non-model plants**.

From the foregoing, it is evidently clear that the cost effective and timely sequencing provided by different NGS technology platforms has impacted positively in advancing the course of non-model plants which earlier had no place in genomics. The technology has enabled scientists to explore the plants to their own benefit and in understanding mechanisms underlying processes of gene expression and secondary metabolism in addition to creation of genomic resources for diversity analysis and marker assisted breeding (Figure 1) through *de novo* analysis which hitherto was impossible due to lack of reference genomes.

The decreasing cost of this technology is however an open door to the possibility of sequencing genomes of individuals of a particular species. This if utilized properly will immensely assist comparative genomics in acquiring vital information about the evolutionary history of non-model plant species by studying the order of their DNA sequences, which had relied on chromosome numbers and ploidy levels. Moreso, protein seq (proteomics) combined with the increasing number of WGS will aid functional genomics in protein identification and consequently perform functional prediction of hypothetical proteins/genes which usually form the largest category during functional (BLASTX) annotations in non-model plants as well as in metabolomics which involves large scale measurements of metabolites level as non-model plants are large repositories of secondary metabolites of economic interest. It will also enable Phenomics for development of large scale phenotypic data for understanding how interactions of genotypes with the environment translate into phenotypic variations in non-model plants. In addition, improvements in these technologies will also advance Bioinformatics in data handling processes.

### Conflict of interest statement

The authors declare that the research was conducted in the absence of any commercial or financial relationships that could be construed as a potential conflict of interest.
