# Supplementary material for: Next Generation Sequencing Technologies: The Doorway to the Unexplored Genomics of Non-Model Plants
Source: Front Plant Sci. 2015 Dec 16;6:1074. doi: 10.3389/fpls.2015.01074 (PMC4679907; doi:10.3389/fpls.2015.01074)
Supplement: Supplementary file 1 [file Table1.DOCX]

**Supplementary Table 1: Summary assessment of the different next generation sequencing technologies with the traditional Sanger method**

| **Platform** | **Sequence Chemistry** | **Template Preparation** | **Run Time (days)** | **Number of Reads** | **Read Length (bp)** | **Cost**  **per Mb**  **(US$)** | **Thorough- put**  **(Mb/day)** | **Merits** | **Demerits** |
| --- | --- | --- | --- | --- | --- | --- | --- | --- | --- |
| **Illumina/ Solexa** | Synthesis/Reversible terminators | Bridge PCR | 3 – 11 | 50 – 200 million | 32-200 | ~ 0.50 | 5000 | High coverage, cost effective and most commonly used | Shorter read lengths, substitution error, less feasible for de novo assembly |
| **454/Roche** | Pyro-  Sequencing | emulsion PCR | 1 | 1 – 1.2 million | 250-700 | 8-20 | 750 | Long reads; fast run time, good choice for de novo assembly | High cost, requires more enzymes, insertion and deletion errors |
| **ABI/SOLiD** | Ligation | emulsion PCR | 8 | ~ 1 billion | 35-85 | ~ 0.50 | 5000 | High accuracy, 2-Base encoding error correction | Short read length, low sequencing speed, longer run time |
| **Helicos** | Reversible terminators | Single molecule | 8 | ~ 1 billion | 32 - 55 | < 0.50 | 5000 | High accuracy, High multi-plexing, no template amplification is needed. | Short read length, high error rate. |
| **Sanger** | Chain terminator | Capillary electrophoresis | 1 | 0.000069 million | 400–1000 | ~ 500 | 6 | Longer read lengths, high accuracy | Requires capillary electrophoresis, high cost, low number of reads |
